# Assessing knowledge of genetics in undergraduate students in Quito, Ecuador

**DOI:** 10.12688/f1000research.18394.2

**Published:** 2019-08-20

**Authors:** David Ortega-Paredes, César Larrea-Álvarez, Michelle Herrera, Esteban Fernandez-Moreira, Marco Larrea-Álvarez

**Affiliations:** 1 Medicine School, Universidad de las Américas, Quito, Udlapark, Via a Nayón, Quito, 170124, Ecuador; 2 Education Unit, Life Science Initiative, Julian Estrella, Quito, 170607, Ecuador; 3 School of Biological Sciences and Engineering, Yachay-Tech University, Hacienda San José, Urcuquí, Imbabura, 100650, Ecuador

**Keywords:** Ecuador, knowledge of genetics, genetic literacy, undergraduate students, survey

## Abstract

Knowledge of genetics is crucial for understanding genetic and genomic tests and for interpreting personal genomic information. Despite this relevance, no data are available about the level of knowledge of genetics in an Ecuadorian population. This investigation sought to survey such knowledge in undergraduate students affiliated with private and public institutions in Quito, the capital city of Ecuador. A total of 350 individuals responded to a validated questionnaire measuring knowledge of genetics. Scores ranged from 45% to 87% (mean: 66.8%), and students achieved slightly better results when asked about genetics and diseases (mean score: 68.3%) than when asked about genetic facts (mean score: 64.9%). Additionally, no significant differences in performance were found among students from private and public institutions. Surprisingly, the lower score obtained (45%) was from a question about how chromosomes are passed to the next generation. The highly educated status of the surveyed population could explain the overall results; nonetheless, the possibility that the correct responses were given by chance cannot be ignored. Therefore, the actual knowledge of genetics among the participants might be different than that revealed by the percentages of correct answers. Consequently, to achieve the goal of ensuring informed decision-making concerning genetic and genomic tests, it seems evident that the national education programs of Ecuador require improvement in the teaching of genetic concepts.

## Introduction

Genetic and genomic testing have transformed our understanding of our health, personal well-being and recreational consumerism. Advances in powerful and cheap genetic analyses have allowed new opportunities to generate information about important conditions, such as cancer, diabetes, and cardiovascular diseases ( Burton, 2015; Perkins *et al.*, 2018; Rafiq *et al*., 2015;; Roberts & Middleton, 2018; Wu *et al.*, 2019). In recent years, access to pharmacogenomics, nutrigenomics, disease risk, ancestry and ethnicity tests, as well as access to sport genetic analyses, has become widespread in low- and middle-income countries. Such genetic and genomic practices are carried out by health care institutions and, moreover, direct-to-consumer (DTC) genetic tests are easily available on the internet ( Covolo *et al.*, 2015; Phillips, 2016). In Ecuador, a case study by the Red Cross found that rape, intimate partner violence and femicide rates are high. Ecuadorian laws offer mothers the right to ask for a free paternity test; a positive result automatically obliges fathers to provide support for their children. Additionally, genetic tests are routine in Ecuador for police investigating rape cases. For these reasons, increasing knowledge about how DNA can be a link between parents and children or between a sexual offender and a crime seems to be a powerful tool for women’s empowerment. Several studies have demonstrated that the understanding and interpretation of personal genomic information is biased by one’s own knowledge and appreciation of basic genetic facts, namely, their level of genetic literacy ( Hooker *et al.*, 2014; Lea *et al.*, 2011; Lontok *et al.*, 2015; Rafiq *et al.*, 2015). Evidently, a basic amount of genetic knowledge is essential to understand and interpret the results of genetic and medical analyses. Therefore, various studies have focused on assessing the impact of knowledge of genetics on perception of genetic facts and understanding of disease onset ( Haga *et al.*, 2013; Hollands *et al.*, 2016; Lea *et al.*, 2011). Unfortunately, despite the obvious necessity to determine knowledge of genetics, to our knowledge there is no available information regarding this matter in our country. Moreover, recent research has shown differences in quality between public and private higher education institutions in Colombia ( Cayon *et al.*, 2017). Therefore, it seems important to assess such differences in Ecuador. The data gathered from these kinds of studies could contribute to the development of programs to reinforce the teaching of genetics to a wider population, which will undoubtedly have a positive impact on national educational programs. Therefore, as a baseline report, we decided to determine the basic knowledge of genetics in undergraduate students in Quito, the capital city of Ecuador. This study provides on the student understanding of genetic concepts and the relation of genetics to disease in a relatively highly educated population based in a developing country. Furthermore, this investigation represents one of the first steps required for building the appropriate strategies to comprehensively assess knowledge of genetics and to ultimately increase the level of genetic literacy in the region.

## Methods

### Setting, recruitment and questionnaire

The main objective of this research was to assess the competence of undergraduate students, who do not follow programs involving biologically related courses (n=350 by convenience sampling method), to respond to a validated survey evaluating a minimum, amount of knowledge about genetics ( Fitzgerald-Butt *et al.*, 2016). This particular questionnaire is suitable at low knowledge levels and was developed for older teenage and young adult patients, along with parents in a pediatric setting. This survey was chosen because the targeted population is not involved in the life sciences/biology area, and, thus, are not prominently exposed to this type of information. Moreover, it appears useful to use an instrument measuring basic knowledge as a baseline report, especially in a region where no information about the competency of students in genetics is available. Surveys were carried out from August to October 2018. Individuals were recruited from 3 public and 4 private institutions located in Quito, the capital city of Ecuador. The identity of the institutions was handled in an anonymous form. The participants were approached at random inside the campuses and asked to fill out a questionnaire consisting of 18 statements, provided in Dataset 1 ( Larrea, 2019), which measured both the actual knowledge of the associations of genetic conditions with diseases and the actual knowledge of genetic facts. For each question, the results are presented as the percentage of correct answers.

### Statistical analysis

Pearson’s chi-square test was used to determine the likelihood that the results (answers) supporting the null hypothesis are not due to chance. Additionally, Student’s *t*-test was used to assess whether the two groups, composed of publicly and privately educated students, presented any significant differences regarding their measure of knowledge about genetics (assuming equal variances). *P* values are reported using a Type I error level of 0.05, 0.01 and 0.001. All data analyses were carried out with MATLAB® version 9.9.9341360 (R2016a). A MATLAB script to repeat the analysis is available in Dataset 2 ( Larrea, 2019).

### Ethics approval

This survey was performed under the format of “common social topics”. Because of the low-risk nature of the study, approval from a committee was not sought. The participants were informed about the objective of the questionnaire; the survey was voluntary and anonymous, and information that could put the person at risk was not collected. All surveyed students provided prior verbal consent. Written consent was not sought from the participants due to the low-risk nature of the study.

## Results

In this research, we present the data gathered as a reference study outlining the knowledge of genetics in undergraduate students. Overall, 350 participants were enrolled in this research (average age: 21.8 years old, SD: ± 2.8); individuals came from diverse backgrounds that did not involve life sciences or medicine. The results varied from 45% to 87% (mean: 66.8%, median: 65%) ( Table 1). The responses to each question can be found in Dataset 3 ( Larrea, 2019). The percentage scores were higher for the subsection regarding the relationship between genetics and the presence of illness (mean: 68.3%). The lower scores within this section were observed when individuals were asked about the inheritance of diseases (mean: 56%, *p*=0.019) and when questioned about the health status of a person carrying an altered gene (mean: 55%, *p*=0.069). The percentage scores were lower for the subsection regarding genetic facts (mean: 64.9%). In particular, the students seemed to have difficulty answering correctly when asked about the quantity of chromosomes present in humans (mean: 58%, *p*=0.004) and about the number of copies of each chromosome passed down to the next generation (mean: 45%, *p*=0.054). In addition to the lower scores, the hypothesis that the questions were answered correctly without any previous knowledge (provided by chance) could not be significantly rejected. Generally, no differences in the overall knowledge of genetics could be found among students enrolled in private and public institutions ( *p*=0.9405). Likewise, no differences between these two groups were observed regarding disease-related questions ( *p*=0.7844) and genetic facts ( *p*=0.7318).

**Table 1.** Knowledge of disease related-concepts and genetic facts of undergraduate students with percentages of correct answers.

	Total population (n=350)		Private institutions (n=170)		Public institutions (n=180)	
Disease-related concepts	% correct	*p-Value ^a^*	% correct	*p-Value ^a^*	% correct	*p-Value* ^a^
1. Some diseases are caused by genes, environment and lifestyle. (T)	87	<0.001	89	<0.001	85	<0.001
2. A gen is a disease. (F)	61	<0.001	63	<0.001	65	<0.001
3. Healthy parents can have a child with an inherited disease. (T)	74	<0.001	76	<0.001	71	<0.001
4. A person with altered (mutated) gene may be completely healthy. (T)	55	0.069	57	0.011	53	0.443
5. All serious diseases are inherited. (F)	56	0.019	54	<0.001	59	0.014
6. The child of a person with an inherited disease will always have the same disease. (F)	58	0.002	58	<0.001	58	0.032
7. Altered (mutated) genes can cause disease. (T)	84	<0.001	88	<0.001	81	<0.001
8. A genetic test can tell you if you have a higher chance to develop a specific disease (T)	80	<0.001	82	<0.001	78	<0.001
Average percentage for this section	68.3		70		68.4	
Genetic facts						
1. You can see a gene with the naked eye. (F)	59	<0.001	58	<0.001	61	0.004
2. Genes are instructions for making proteins, which help the body grow and work properly. (T)	57	0.008	61	0.004	54	0.357
3. A gene is a piece of DNA. (T)	77	<0.001	74	<0.001	80	<0.001
4. Genes are inside cells. (T)	69	<0.001	71	<0.001	71	<0.001
5. A chromosome contains many genes. (T)	78	<0.001	77	<0.001	79	<0.001
6. Genes determine traits such as height, eye color and facial appearance. (T)	84	<0.001	82	<0.001	86	<0.001
7. A person has thousands of genes. (T)	73	<0.001	74	<0.001	73	<0.001
8. Identical twins have different sets of genes. (F)	49	0.915	47	0.307	53	0.443
9. Humans have 20 pairs of chromosomes. (F)	58	0.004	53	0.027	61	0.004
10. Parents pass both copies of each chromosome to their child. (F)	45	0.054	49	0.610	41	0.014
Average percentage for this section	64.9		63.8		68.6	
Overall average percentage	66.8		66.6		67	

^a^ *p-*values for determining answers provided by chance were calculated using Pearson’s Chi squared test. T, true; F, false.

## Discussion

In this report, we portray the percent of correct answers to an 18-item questionnaire measuring a minimum amount of knowledge about genetics. Overall, this Andes-located population of undergraduate students demonstrated some basic knowledge toward genetic concepts and their relation to diseases. Nonetheless, student knowledge on facts about genetic proved to be less strong. This tendency was observed in both privately and publicly educated individuals with no significant difference. These results are lower in comparison to the published reports on general populations that have made extensive use of similar survey instruments to determine knowledge about genetics. For instance, Haga & colleagues (2013) found higher scores in a general population based in the US. However, similar scores to those reported here were found by Jallinoja & Aro (1999) in a study performed on a general population in Finland. Furthermore, a group composed of adolescents and young adults suffering from congenital heart disease scored similar results ( Fitzgerald-Butt *et al.*, 2016). Notably, the present results are somewhat higher than those obtained from a Dutch population suffering from asthma, diabetes mellitus type II and cardiovascular disease ( Calsbeek *et al.*, 2007). It is evident that demographic differences may account for the variances in the results. Nevertheless, these results may also imply notable differences between Ecuadorian, US and European science and health education programs ( Lontok *et al.*, 2015). The lowest scores obtained were for the two questions involved in how chromosomes are passed down to the next generation. These outcomes might be related to the hitherto reported conceptual variation in biology textbooks, which have been shown to have detrimental repercussions regarding the students understanding of conceptual knowledge, models in particular, within the context of genetics ( Gericke *et al*., 2014; Gericke *et al*., 2013). This means that students may not understand the power of genetics to address important issues for the Ecuadorian population, such as determining paternity, solving crimes or understanding our ethnic genetic background. To the best of our knowledge, this study is the first to report a measurement of knowledge of genetics in an Ecuadorian population.

Additionally, the presented results indicate that the probability of participants providing correct responses by chance could not be significantly discarded ( Table 1). This fact implies that the actual knowledge might be different from the one asserted by the percentages of correct answers. Therefore, to have a better understanding on the actual knowledge, we suggest to implement, for each question, a section in which the interviewed is asked to provide a degree of certainty for his or her answer. Indeed, such an analysis has been applied to measure diabetic patients’ knowledge about the illness, and was shown to be useful in determining more efficaciously their degree of mastery about the subject ( Leclercq, 2010). Individuals affiliated with private and public universities responded with similar accuracy. The observed average scores might reflect the high level of education of the respondents. It is worth mentioning that the interviewed people did not follow any biologically/medically related courses. Furthermore, this study provides adequate estimates of the knowledge of genetic and its relation to disease in a non-specialized population. It is important to note that the participants’ knowledge may not be as strong as it appears. As mentioned earlier, the scores do not differ substantially from the earlier studies making use of similar surveys. Nonetheless, the scores were lower than those obtained from a study performed in the US ( Haga *et al.*, 2013) where genetic education is constantly improving ( Lontok *et al.*, 2015). Based on these observations, a revision of the genetic content covered in educational programs and the implementation of science popularization initiatives seem imperative.

Some limitations of this study should be mentioned. This investigation did not attempt to address the differences in knowledge of genetics among groups classified by characteristics such as sex, ethnic group, age, family history of inherited diseases or level of education. Instead, this study was intended to be focused solely on a general undergraduate population not studying biology or medicine. Furthermore, more universities in different cities should be sampled to have a national perspective on students’ insights about genetics. Overall, these results provide a glimpse of the students’ standpoint toward genetics and its involvement in disease. Nevertheless, more effort is decisively needed to design and execute plans that will ensure an optimized method to measure knowledge of genetics in a larger and more diverse population. The data generated using these approaches will be proven essential when designing educational programs involving genetics and health. The application of such programs will be fundamental for the general population to avoid misunderstandings about genetics and to avoid the incorrect utilization of scientific terms.

Follow-up studies will try to explore the knowledge about genetics and the attitudes toward related subjects, including genetic testing, stem cells, regenerative medicine and genetically modified organisms (GMOs). The expected results will provide improved insight into the population’s knowledge and will serve as a foundation for developing better strategies to increase the level of genetic literacy in our community.

## Data availability

### Extended data

Open Science Framework: Assessing genetic knowledge in Ecuador. https://dx.doi.org/10.17605/OSF.IO/ZUVMN ( Larrea, 2019)

This project contains the following extended data:

- Dataset 1.pdf (the questionnaire in the original Spanish language and its translation into English)
- Dataset 2.pdf (the MATLAB script to reproduce the analysis)

### Underlying data

Open Science Framework: Assessing genetic knowledge in Ecuador. https://dx.doi.org/10.17605/OSF.IO/ZUVMN ( Larrea, 2019)

This project contains the following underlying data:

- Dataset 3.csv (a spreadsheet containing all responses to the evaluation)
