# Supplementary material for: Assessing knowledge of genetics in undergraduate students in Quito, Ecuador
Source: F1000Res. 2019 Aug 20;8:290. Originally published 2019 Mar 14. [Version 2] doi: 10.12688/f1000research.18394.2 (PMC6719661; doi:10.12688/f1000research.18394.2)
Supplement: Peer review history

# Peer review: Reviewer response for version 2

**DOI:** 10.5256/f1000research.21828.r52763

**Published:** 2019-09-02

**Authors:** Rebecca Carver

**Affiliations:** 1 Department of Communication, Norwegian Institute of Public Health, Oslo, Norway

I have now read the revised version and the authors have made appropriate changes.

I confirm that I have read this submission and believe that I have an appropriate level of expertise to confirm that it is of an acceptable scientific standard.

# Peer review: Reviewer response for version 1

**DOI:** 10.5256/f1000research.20127.r47624

**Published:** 2019-05-21

**Authors:** Rebecca Carver

**Affiliations:** 1 Department of Communication, Norwegian Institute of Public Health, Oslo, Norway

This study provides valuable insight into the level of knowledge of genetics in an Ecuadorian population.

**Introduction**

 Be careful with terminology regarding “perspectives” towards genetics, as this is not the same as knowledge about genetics. The same goes for “attitudes”.

**Study design**

 The first sentence in the methods section does not make sense. How is this a “baseline report on the attitude among undergraduate students toward genetic concepts?” What do the authors mean? Knowledge is not the same as attitudes.

 The authors of the chosen instrument (Fitzgerald-Butt et al 2016 ^1^) have stated in their paper that the instrument can be used for older teenage and young adult patients and parents in the pediatric setting, and that is most informative for individuals with below average genetic knowledge. How is this instrument applicable to the current study’s Ecuadorian student population, which is a very different sample population than the one for which the instrument was developed? What were the reasons for choosing this particular instrument? Can the authors justify their choice of instrument?

**Results**

 What percentage of correct answers is “adequate” in the chosen instrument? Is there a reference value in the original instrument that can used to compare the results of this survey? (E.g. what minimum percentage of answers should the respondents answer correctly in order to have an adequate amount of knowledge?).

**Discussion**

 The results show that students achieved slightly better results when asked about genetics and diseases than when asked about genetic facts. One possible explanation for this could be that the questions about disease relate more to people’s lives than genetic facts. The authors state that they were surprised by the lower score obtained on a question about how chromosomes are passed to the next generation, but students may regard this type of “textbook knowledge” as less relevant to their everyday lives, and thus be less inclined to remember it. There is a lot of literature on how the genetics content in textbooks is lagging behind modern scientific developments – e.g. see previous literature by Gericke et al. and Dougherty et al. – and this may be an explanation for the results in this study. The authors might like to comment on this.

**Conclusions**

 The authors conclude by saying that despite the relatively adequate score overall (66.8% correct answers), the possibility that the correct responses were given by chance cannot be ignored – and that the actual knowledge of genetics among the participants might be less that that revealed by their answers. Consequently, the authors assert; “it seems evident that the national education programs of Ecuador require improvement in teaching of genetic concepts”. However, if the responses were given by chance, is it not also possible for the actual knowledge of genetics among the participants to be *higher* than that revealed here? Could the authors comment on this?

I confirm that I have read this submission and believe that I have an appropriate level of expertise to confirm that it is of an acceptable scientific standard, however I have significant reservations, as outlined above.

## References

[1] : Measuring genetic knowledge: a brief survey instrument for adolescents and adults. *Clin Genet*.2016;89(2) : 10.1111/cge.12618 235-43 10.1111/cge.12618 26032340PMC5215811

# Peer review: Reviewer response for version 1

**DOI:** 10.5256/f1000research.20127.r47621

**Published:** 2019-05-07

**Authors:** Vasiliki Mollaki

**Affiliations:** 1 Hellenic National Bioethics Commission, Athens, Greece

The study aimed to assess the knowledge of genetics in a selected population of students from both public and private institutions.

 The work is described in sufficient detail (regarding the samples size, study design and statistical analysis). The authors acknowledge the study limitations.

 Although similar studies have been performed in other countries, this is the first study assessing genetic knowledge in Ecuador. The result of the study could be proved useful for policy makers and educational programs.

I confirm that I have read this submission and believe that I have an appropriate level of expertise to confirm that it is of an acceptable scientific standard.
